# Gene Expression Analysis of the Pleiotropic Effects of TGF-β1 in an *In Vitro* Model of Flexor Tendon Healing

**DOI:** 10.1371/journal.pone.0051411

**Published:** 2012-12-10

**Authors:** Youssef M. Farhat, Alaa A. Al-Maliki, Tony Chen, Subhash C. Juneja, Edward M. Schwarz, Regis J. O’Keefe, Hani A. Awad

**Affiliations:** 1 Department of Biomedical Engineering, University of Rochester, Rochester, New York, United States of America; 2 The Center for Musculoskeletal Research, University of Rochester Medical Center, Rochester, New York, United States of America; 3 Department of Orthopaedics, University of Rochester Medical Center, Rochester, New York, United States of America; Ohio State University, United States of America

## Abstract

Flexor tendon injuries are among the most challenging problems for hand surgeons and tissue engineers alike. Not only do flexor tendon injuries heal with poor mechanical strength, they can also form debilitating adhesions that may permanently impair hand function. While TGF-β1 is a necessary factor for regaining tendon strength, it is associated with scar and adhesion formation in the flexor tendons and other tissues as well as fibrotic diseases. The pleiotropic effects of TGF-β1 on tendon cells and tissue have not been characterized in detail. The goal of the present study was to identify the targets through which the effects of TGF-β1 on tendon healing could be altered. To accomplish this, we treated flexor tendon tenocytes cultured in pinned collagen gels with 1, 10 or 100 ng/mL of TGF-β1 and measured gel contraction and gene expression using RT-PCR up to 48 hours after treatment. Specifically, we studied the effects of TGF-β1 on the expression of collagens, fibronectin, proteoglycans, MMPs, MMP inhibitors, and the neotendon transcription factors, Scleraxis and Mohawk. Area contraction of the gels was not dose-dependent with the TGF-β1 concentrations tested. We observed dose-dependent downregulation of MMP-16 (MT3-MMP) and decorin, and upregulation of biglycan, collagen V, collagen XII, PAI-1, Scleraxis, and Mohawk by TGF-β1. Inter-gene analyses were also performed to further characterize the expression of ECM and MMP genes in the tenocyte-seeded collagen gels. These analyses illustrate that TGF-β1 tilts the balance of gene expression in favor of ECM synthesis rather than the matrix-remodeling MMPs, a possible means by which TGF-β1 promotes adhesion formation.

## Introduction

Each year, millions of Americans injure their hands in the workplace, home and elsewhere resulting in significant morbidity and thousands of lost workdays [1], [2]. Among these injuries, flexor tendon lacerations remain a challenge for surgeons and tissue engineers alike. While flexor tendons heal with reduced mechanical strength, the most clinically significant issue is the formation of adhesions that impair hand function in as many as 30–60% of cases regardless of surgical approach [3], [4]. Despite the human and economic impact of this problem, there are presently no pharmacologic or biologic agents available for the prevention of tendon adhesions [5]. In fact, the only accepted means of preventing adhesion formation in flexor tendons remains physical therapy [5], but even with physical therapy, the strength of healed tendons is markedly less than prior to injury, and debilitating adhesions have been reported to occur in as many as 50% of zone II flexor tendon injuries [6].

Evidence exists that flexor tendon adhesions [7], [8], [9] and scarring in other parts of the body [10], [11], [12] are orchestrated primarily by the growth factor, Transforming Growth Factor Beta 1 (TGF-β1). One of the characteristics of TGF-β1 mediated healing is the replacement of native extracellular matrix proteins with that of a fibrous scar, consisting mostly of fibronectin, collagen I and collagen III [13]. Antagonism of TGF-β1 has been reported to reduce scarring and/or adhesion formation in animal models of tendon [7], [8], [9], abdominal [10], and skin [11], [12] injury and repair. Unfortunately, antagonizing TGF-β1 also led to the loss of mechanical strength within healing tendon [8], [9], suggesting that TGF-β1 mediated matrix production is essential to the strength of the repair.

The ultimate goal of flexor tendon repair is to restore the mechanics of the tendon to that of its uninjured state. The composition and organization of the extracellular matrix (ECM) of uninjured tendon is what defines its mechanical strength and behavior. The major component of tendon ECM is collagen, a rope-like protein which forms several levels of hierarchical structures called fibrils, fibers and fascicles (reviewed in [14]). Collagen I is the most abundant collagen in normal tendon followed by collagen III [15]; however, collagen III and fibronectin are highly upregulated during tendon healing [16]. During development, collagens V and XII as well as the proteoglycans Decorin, Lumican and Biglycan, are thought to regulate the process of *fibrillogenesis* (reviewed in [17]), where collagen molecules coalesce and assume their highly ordered structure. Given TGF-β1’s association with scar tissue and adhesion formation, we hypothesized that TGF-β1 would upregulate ECM proteins associated with scarring and fibrosis [18], including fibronectin, collagen I, and collagen III. On the other hand, we hypothesized that TGF-β1 would either downregulate or have no effect on the ECM components associated with tendon development, such as collagen V, collagen XII, biglycan, decorin and lumican.

The condition of the ECM in developing, normal, and healing tissue is both a function of ECM production and degradation. Matrix metalloproteinases (MMPs) are the class of proteinases that are best known for their ability to degrade various ECM components, including collagen, fibronectin, and proteoglycans [19]. Hence, MMPs are involved in a number of processes throughout the body, including development [19] and wound healing [20]. MMP-2, MMP-3 and MMP-14 (also known as membrane type 1 matrix metalloproteinase or MT1-MMP) are expressed in normal tendon and upregulated during tendon healing [21], [22]. MMP-16 (MT3-MMP), on the other hand, plays an essential role in developing tendon by regulating the activation of MMP-2, and, in turn, causing the maturation of tendon fibrils [19]. Given their role in the development, degradation and remodeling of tissue, we hypothesized that TGF-β1 inhibits MMP expression and/or activity and thereby interrupts key regenerative processes as well as causes the persistence of adhesions and scarring. To this end, we investigated the effects of TGF-β1 on the expression of the aforementioned MMPs as well as their inhibitors, Plasminogen Activator Inhibitor 1 (PAI-1, or *Serpine1*) and Tissue Inhibitor of Metalloproteinase 2 (TIMP-2).

It is also theorized that regenerative, scarless healing of force transmitting tendons would require the activation of Scleraxis (*Scx*) and Mohawk (*Mkx*); transcription factors that are highly upregulated during tendon development [23], [24], [25], [26]. This is supported by findings that both Scleraxis [27] and Mohawk [25] regulate the expression of ECM genes in tendon cells. While it has been shown that TGF-β1 maintains Scleraxis expression in developing tendon [28], the dose-dependent effects of TGF-β1 on Scleraxis expression in adult tendon cells have not been reported previously. In addition, the effect of TGF-β1 on Mohawk expression has never been evaluated to our knowledge. Given the association of TGF-β1 with scar formation and lack of proper tissue regeneration throughout the body [7], [11], [29], we initially hypothesized that TGF-β1 may downregulate one or more of these important developmental genes in adult tendon cells, resulting in tendon scarring and adhesions rather than tendon regeneration.

Collagen hydrogels are a desirable model of tendon healing, because collagen is the primary constituent of tendon [14], and 95% of the collagen in tendon is collagen type I [15]. These pinned gels provide cultured cells with a three-dimensional extracellular matrix environment that they can reproducibly remodel and contract when stimulated appropriately with growth factors, such as TGF-β1 [30], [31]. The contraction of collagen gels is thought to be analogous to the ECM remodeling observed in wound healing [32] and during the formation of tendon adhesions [33]. The degree of contraction depends on the concentration of purified collagen used [34], the cell-seeding density [35], and the concentration of growth factors in the culture media [31]. In addition, tenocyte-seeded collagen hydrogels generally have a high cell/collagen ratio that mimics the provisional granulation matrix formed early during the healing process, and whose aberrant remodeling in response to factors such as TGF-β1 might lead to adhesions. This is a distinct advantage over monolayer cell culture systems classically used in similar experiments.

**Figure 1:**
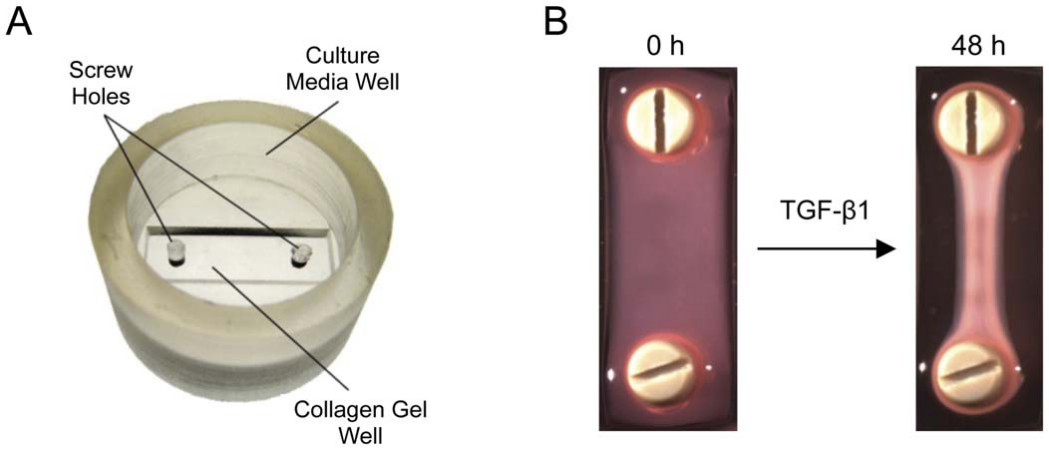
*In vitro* tendon healing model used to assess the effects of TGF-β1 on tenocyte gene expression. (A) For each experiment, tenocyte-seeded collagen was cast into the collagen gel well of a custom culture construct between two screws. After gelation, 2 mL of media was added to the culture media well. (B) Tenocyte-seeded collagen gels treated with TGF-β1 contracted over 48 hours and aligned themselves between two screws, forming a tissue that grossly resembled tendon.

For the present experiments, we designed silicone constructs that fit into 6-well plates and accommodate approximately 450 µL of cell-seeded collagen I, which is gelled between two polymer screws (Figure 1A). The screws act as posts around which the collagen can contract, allowing fibrils to orient in a single direction (Figure 1B), more closely resembling tendon microstructure than monolayer cultures or models with free-floating or anchored collagen gels. Using this *in vitro* model of tendon healing, we investigated the dose-dependent effects of TGF-β1 (1, 10 or 100 ng/mL) on flexor tendon tenocytes cultured in pinned collagen hydrogels. We opted to evaluate this range of TGF-β1 concentrations based on previous reports that investigated the effects of a range of TGF-β1 concentrations on the contraction of collagen gels by Tenon’s capsule fibroblasts [31]. Their study showed that significant contraction of 3D collagen gels began at a minimum concentration of 10^−11^ M (∼1 ng/mL of TGF-β1) and peaked at 10^−9^ M (∼100 ng/mL). Our experimental readouts were collagen gel area contraction and the relative expression of the aforementioned genes that are involved in the development, regulation, and healing of tendon.

## Results

### TGF-β1 Increased Collagen Gel Contraction Over 48 Hours

**Figure 2:**
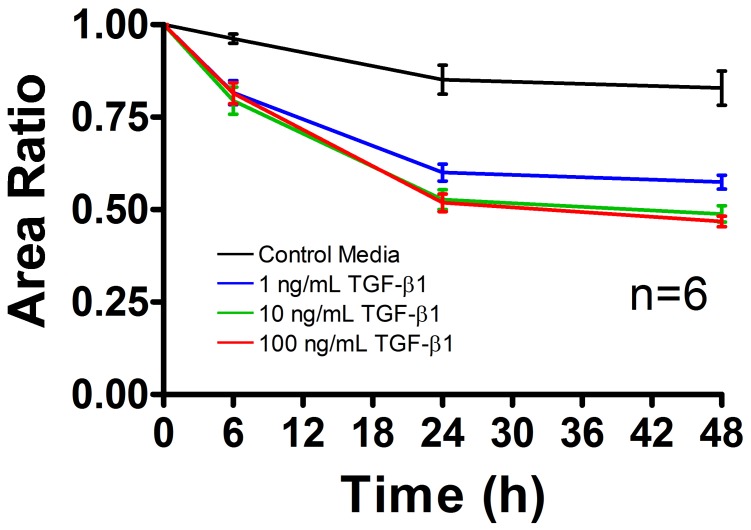
rea contraction of the collagen gels as a functional measure of TGF-β1 activity. Digital images of tenocyte-seeded collagen gels treated with control media (containing 1% FBS and 1% Pen Strep) supplemented with 0, 1, 10 or 100 ng/mL of TGF-β1 were analyzed using ImageJ. The area ratio (gel area divided by the area at 0 hours) was determined at 0, 6, 24 and 48 hours after treatment to assess contraction. Gels treated with 1–100 ng/mL of TGF-β1 contracted significantly more than controls after only 6 hours (p<0.001). No differences in area ratio were observed between the three doses of TGF-β1 at any time except in gels treated with 1 vs. 100 ng/mL at 48 hours (p<0.05). N = 6 gels per treatment per time point. Error bars represent the standard error of the mean (SEM).

Gel contraction at 0, 6, 24 and 48 hours after treatment was calculated based on surface area in order to evaluate the degree of cellular remodeling elicited by the three doses of TGF-β1 (Figure 2). Gels treated with control media (MEM α containing 1% FBS and 1% Pen Strep) contracted minimally over the course of 48 hours to only 83% of their initial area. Gels treated with 1, 10 and 100 ng/mL of TGF-β1, on the other hand, contracted to an average of 57%, 49% and 47% of their original area, respectively, after 48 hours. TGF-β1 treated gels showed significant differences in contraction from control gels after only 6 hours (p<0.001). There were no dose-dependent differences between gels treated with TGF-β1 at any time point except between gels treated with 1 ng/mL and those treated with 100 ng/mL at 48 hours (p<0.05).

### TGF-β1 Increased the Expression of Fibronectin and Collagen Types I, III, V and XII

**Figure 3:**
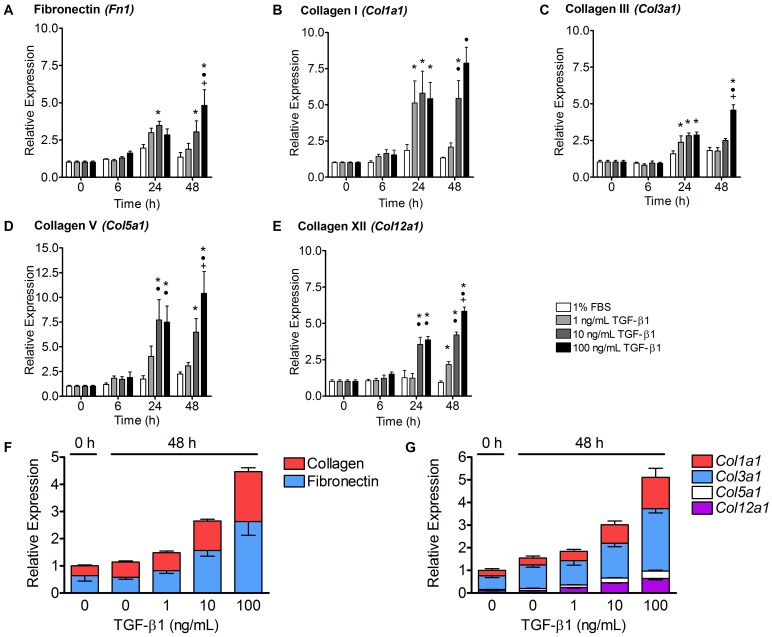
TGF-β1 increased the expression of fibronectin and collagen genes. (A–E) The mean expression (± SEM) of individual fibronectin and collagen genes in tenocyte-seeded collagen gels after treatment with control media supplemented with 0–100 ng/mL of TGF-β1 over 48 hours was assessed with RT-PCR. Only collagens I, III and XII were upregulated significantly by the lowest dose, 1 ng/mL of TGF-β1. On the other hand, all of the ECM genes were upregulated by 10 and 100 ng/mL doses of TGF-β1 at 24 or 48 hours. The highest dose, 100 ng/mL, had the longest-lasting effects and caused the greatest upregulation of all genes at 48 hours. N = 5−6 gels per treatment per time point. *p<0.05 vs. control media, •p<0.05 vs. 1 ng/mL TGF-β1, +p<0.05 vs. 10 ng/mL TGF-β1. (F) Inter-gene analysis of fibronectin and collagen expression (± SEM) before (0 hours) and 48 hours after treatment with 0–100 ng/mL TGF-β1 normalized to 0 hours. The expression of all four collagens combined (red) was approximately equal to the expression of fibronectin (blue) at each time point and treatment condition. (G) Inter-gene analysis of the expression levels of individual collagen isoforms (± SEM) normalized to 0 hours. Collagen III was the most highly expressed isoform in tenocytes cultured in collagen gels before and after TGF-β1 treatment. Collagen I was the next most expressed isoform. The other two isoforms, collagen V and XII, each represented only 4–12% of the total collagen transcripts analyzed, but their expression increased substantially after treatment with 100 ng/mL of TGF-β1 to levels similar to the expression of collagens I and III before treatment (0 hours).

Real-Time Polymerase Chain Reaction (RT-PCR) was performed on tenocyte-seeded collagen gels at 0, 6, 24 and 48 hours after treatment to evaluate the effects of TGF-β1 on gene expression. The gene expression of the extracellular matrix proteins, fibronectin and collagen types I, III, V and XII increased in gels treated with TGF-β1 (Figure 3, A–E). The lowest dose of TGF-β1, 1 ng/mL, stimulated significant, 2.5- and 5-fold increases in collagens I and III, respectively, at 24 hours (p<0.05) and in collagen XII at 48 hours (p<0.01) compared to control media, but had no significant effect at any other time point among the fibronectin and collagen genes analyzed. In contrast, 10 and 100 ng/mL of TGF-β1 stimulated more robust 5–10 fold increases in almost every fibronectin and collagen gene assessed at 24 and 48 hours after treatment. Interestingly, while the 1 and 10 ng/mL TGF-β1 groups showed few changes in expression from 24 to 48 hours, the 100 ng/mL treatment group showed a steady increase in upregulation of ECM gene expression throughout the experiment, with the highest level of expression at 48 hours.

A novel inter-gene analysis was performed to compare the expression of fibronectin and collagen types I, III, V and XII in tenocyte-seeded collagen gels before (0 hours) and 48 hours after treatment with TGF-β1. First, the total expression of all four collagens combined (Figure 3F, red) was compared to the expression of fibronectin (blue). Fibronectin was highly expressed by the tenocyte-seeded collagen gels, with transcript levels slightly greater than or equal to those of all four collagen genes combined (Figure 3F). Both collagen and fibronectin genes increased steadily with increasing doses of TGF-β1, roughly preserving the expression ratio between the two matrix proteins.

We also compared the relative transcript levels of the four collagen types analyzed at 0 and 48 hours for each treatment group (Figure 3G). Interestingly, collagen III expression (blue) was most highly expressed at baseline and at 48 hours of treatment with or without TGF-β1. Collagen I (red) was the second most highly expressed type, and it increased substantially after treatment with 100 ng/mL of TGF-β1. The other two types, collagens V (white) and XII (purple), accounted for a small percentage of the total collagen transcripts at 0 hours (about 4% and 12%, respectively), but after treatment with TGF-β1, there was a dose-dependent increase in their expression at 48 hours.

### TGF-β1 Increased the Expression of Biglycan, but not Decorin

**Figure 4:**
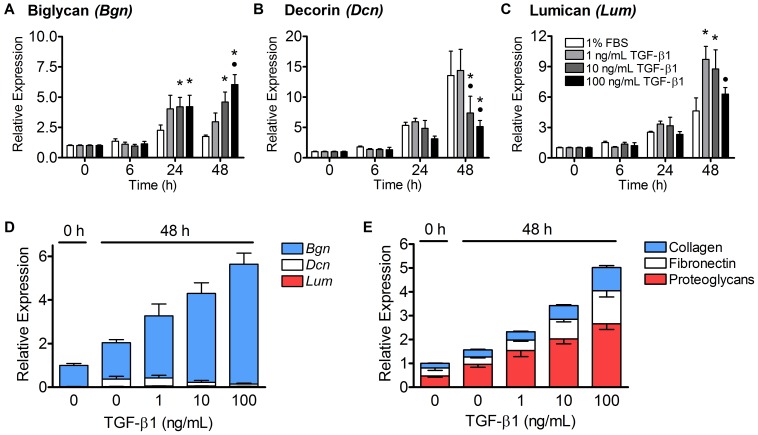
TGF-β1 increased the expression of biglycan and decreased the expression of decorin. (A–C) The mean expression (± SEM) of proteoglycan genes coding for biglycan, decorin, and lumican was evaluated in tenocyte-seeded collagen gels after treatment with control media or 1–100 ng/mL of TGF-β1 over 48 hours. Biglycan expression was significantly increased in the 10 and 100 ng/mL treatment groups at 24 and 48 hours (p<0.05, Panel A). Decorin expression, on the other hand, increased 14-fold in the control and 1 ng/mL treatment groups, but this increase was suppressed by 10 and 100 ng/mL of TGF-β1 (p<0.05, Panel B). Lumican expression was significantly increased with 1 and 10 ng/mL TGF-β1 (p = 0.001), but not 100 ng/mL TGF-β1 (Panel C). N = 5−6 gels per treatment per time point. *p<0.05 vs. control media, •p<0.05 vs. 1 ng/mL TGF-β1. (D) Inter-gene analysis of the proteoglycans revealed that biglycan expression was the highest at all treatments and time points (82–98%), followed by decorin (2–17%) and lumican (<2%). (E) The relative transcript levels of the proteoglycans, fibronectin, and collagen were also determined. At each treatment and time point, proteoglycans (red) were expressed most highly, followed by fibronectin (white) and collagen (blue). All categories of ECM genes were upregulated in a dose-dependent manner by 1–100 ng/mL of TGF-β1 at 48 hours.

The expression of the small leucine-rich proteoglycans (SLRPs), biglycan, decorin, and lumican, in tenocyte-seeded collagen gels treated with control media or 1–100 ng/mL of TGF-β1 was also assessed with RT-PCR (Figure 4, A–C). While all three doses of TGF-β1 increased biglycan gene expression at 24 and 48 hours, only the 10 and 100 ng/mL treatment groups were significantly different from controls (p<0.05). Decorin, on the other hand, showed increases in expression of up to 15-fold in the control media and 1 ng/mL TGF-β1 groups over the course of the 48 hour experiment, but this time-dependent increase was suppressed in gels treated with 10 and 100 ng/mL TGF-β1 (p<0.05). Lumican expression also increased 4.5-fold over the course of the experiment in gels treated with control media, and this increase was significantly enhanced by 1 and 10 ng/mL of TGF-β1 (p<0.001).

An inter-gene analysis was used to assess the relative expression of biglycan, decorin and lumican in the tenocyte-seeded collagen gels before and 48 hours after treatment with TGF-β1 (Figure 4D). Biglycan expression was the highest at all treatments and time points (82–98%), followed by decorin (2–17%) and lumican (<2%). The ratio of biglycan to decorin was decreased from 50 at 0 hours to only 5 in the control media at 48 hours, because decorin expression was significantly increased in that group. However, treatment with 10 and 100 ng/mL of TGF-β1 raised the ratio of biglycan to decorin expression to 24 and 52, respectively, as a result of increased biglycan and decreased decorin expression in the 100 ng/mL treatment group.

The relative transcript levels of proteoglycans, fibronectin and collagen were similarly evaluated (Figure 4E). Proteoglycan expression (red) made up 47–68% of the ECM transcripts at all treatments and time points, with fibronectin (white) and collagen (blue) composing 19–34% and 16–20%, respectively, of the remaining expression. Qualitatively, all three categories of ECM genes increased with 1–100 ng/mL of TGF-β1 in a dose-dependent manner at 48 hours.

### TGF-β1 Decreased MMP-16 Expression but had Little Effect on Other MMPs

**Figure 5:**
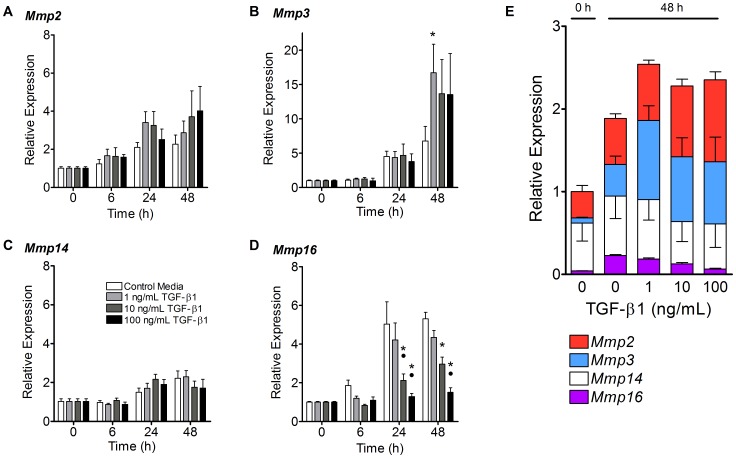
TGF-β1 had little effect on MMP-2, MMP-3 and MMP-14 expression, but decreased expression of MMP-16. (A–D) The mean expression (± SEM) of MMP genes were evaluated in tenocyte-seeded collagen gels after treatment with control media or 1–100 ng/mL of TGF-β1 over 48 hours. MMP-2 (A) and MMP-14 (C) increased 1.5- to 4-fold over 48 hours, but were not significantly affected by treatment with 1–100 ng/mL of TGF-β1. While TGF-β1 treated gels expressed about twice as much MMP-3 compared to control gels at 48 hours, only the 1 ng/mL TGF-β1 treatment group reached significance (p<0.01, Panel B). MMP-16 expression, on the other hand, increased 4- to 5-fold at 24 and 48 hours in the control and 1 ng/mL groups, but this increase was significantly reduced in the 10 and 100 ng/mL TGF-β1 treated gels (p<0.01, Panel D). N = 5−6 gels per treatment per time point. *p<0.01 vs. control media, •p<0.01 vs. 1 ng/mL TGF-β1. (E) Inter-gene analysis of the expression of the MMPs before and 48 hours after treatment with TGF-β1. At 0 hours, MMP-14 expression was roughly equal to the other MMPs combined; however, after 48 hours, the levels of MMP-2 and MMP-3 increased in all groups regardless of the presence of TGF-β1. MMP-16 constituted the smallest portion of MMP expression in all treatment groups and time points.

Although TGF-β1 had only a small effect on the expression of MMP-2, MMP-3, and MMP-14, it resulted in a marked dose-dependent reduction in the gene expression of MMP-16 in a dose-dependent manner (Figure 5A–D). Interestingly, TGF-β1 did not seem to have an effect on the expression of MMP-2 and MMP-14, which increased 1.5- to 4-fold in all treatment groups over the course of 48 hours. Similarly, MMP-3 expression was upregulated 6–15 fold over the course of the experiment. While the highest levels of MMP-3 expression were observed in the TGF-β1 treated gels at 48 hours, only the 1 ng/mL TGF-β1 achieved a significant increased compared to the control group (p<0.01). MMP-16 expression increased five-fold in the control media treated gels at 24 and 48 hours, and TGF-β1 appeared to suppress that increase in a dose-dependent manner. Specifically, 10 and 100 ng/mL TGF-β1 caused a significant reduction in MMP-16 expression at 24 and 48 hours compared to control media and 1 ng/mL TGF-β1 at 24 and 48 hours (p<0.01).

An inter-gene analysis of the relative levels of MMPs was performed (Figure 5E). At 0 hours, the expression of membrane-bound MMP-14 constituted 58% of total MMP expression, followed by MMP-2 (32%), MMP-3 (6%), and MMP-16 (4%). However, after 48 hours, the levels of the secreted MMPs, MMP-2 and MMP-3, increased in all groups regardless of the presence of TGF-β1, and their combined expression surpassed MMP-14 expression in all treatment groups. At all time points, membrane-bound MMP-16 constituted the smallest portion of MMP expression. Interestingly, in gels treated with control media, MMP-16 transcripts increased from 4% to 12% of the total MMPs expressed by the tenocyte-seeded gels. However, TGF-β1 treatment totally reversed this trend and reduced MMP-16 levels to only 3% in the 100 ng/mL group at 48 hours.

### TGF-β1 Increased PAI-1 Expression but did not Affect TIMP-2 Expression

**Figure 6:**
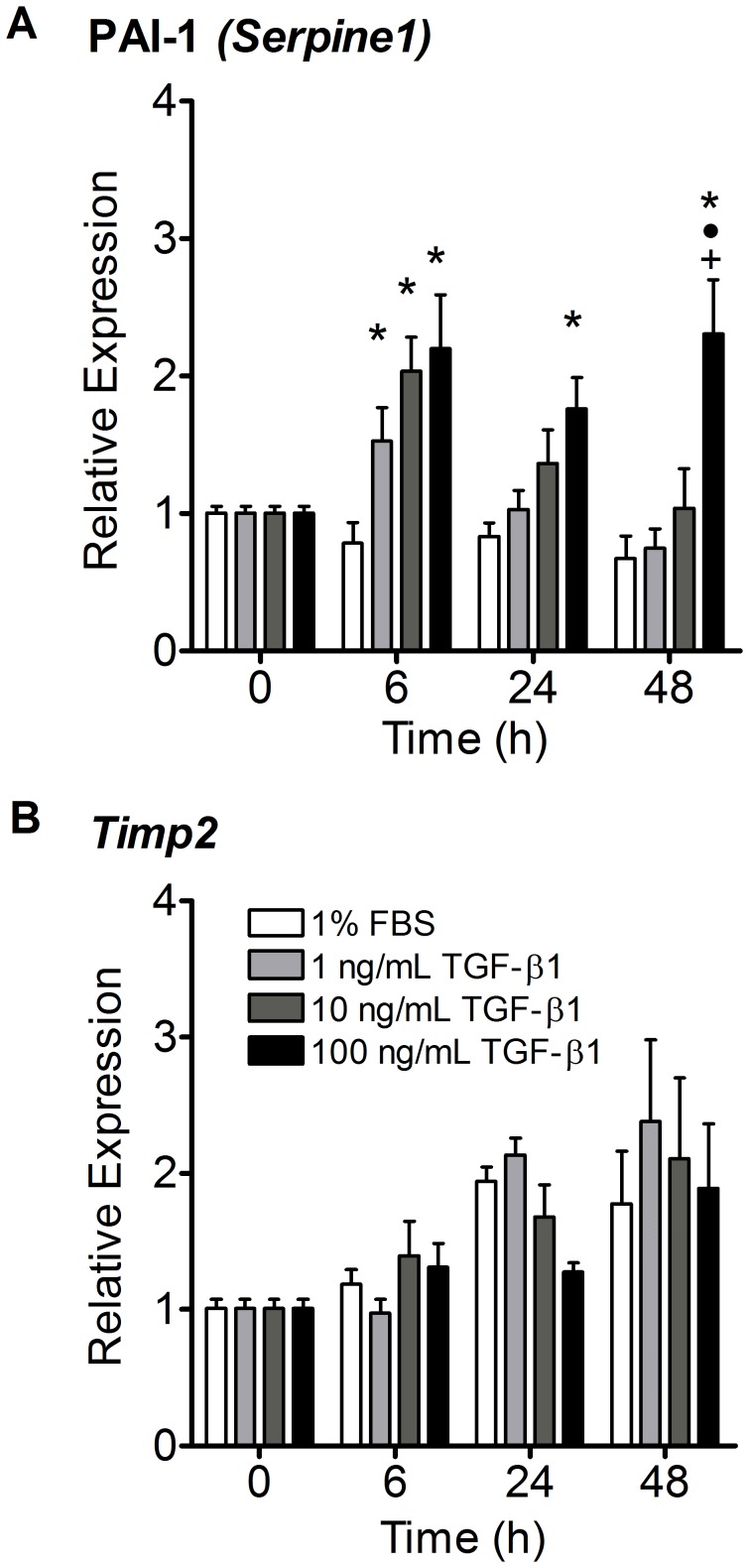
TGF-β1 did not affect TIMP-2 expression, but upregulated PAI-1. (A) PAI-1 responded to all three doses of TGF-β1 with significant upregulation at as early as 6 hours (p<0.05). However, only the 100 ng/mL dose of TGF-β1 appeared to have sustained effects on PAI-1 expression at 24 and 48 hours. (B) TIMP-2 expression increased about 2-fold in all treatment groups over 48 hours and was not significantly affected by TGF-β1 at any concentration or time point tested. N = 5−6 gels per treatment per time point. *p<0.05 vs. control media, •p<0.001 vs. 1 ng/mL TGF-β1, +p<0.001 vs. 10 ng/mL TGF-β1.

PAI-1 expression was upregulated approximately 2-fold after only 6 hours by all three doses of TGF-β1 (p<0.05 - Figure 6A). The upregulation of PAI-1 was short-lived in the 1 and 10 ng/mL TGF-β1 treatment groups, but was sustained up to 48 hours after treatment in the 100 ng/mL TGF-β1 treated gels. While gene expression of the MMP-inhibiting protein, TIMP-2, increased 1.5 to 2 fold throughout the duration of the experiment, its expression was not significantly affected by the presence of TGF-β1 (Figure 6B).

### TGF-β1 Highly Upregulated Scleraxis and Mohawk Expression

**Figure 7:**
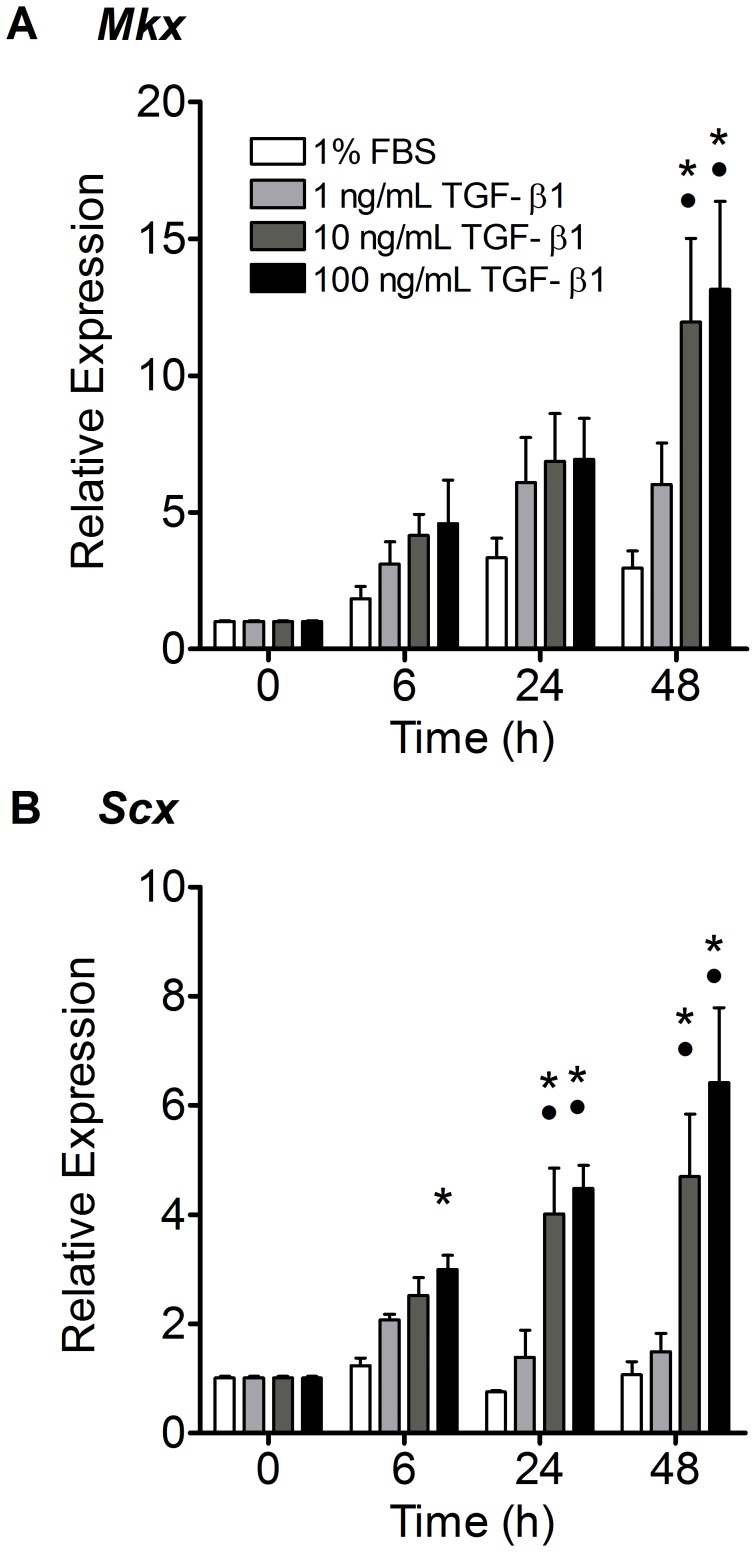
TGF-β1 highly upregulated Mohawk (*Mkx*) and Scleraxis (*Scx*), genes necessary for tendon development. (A) Mohawk was significantly upregulated by 10 and 100 ng/mL TGF-β1 at 48 hours. (B) Scleraxis was upregulated by 100 ng/mL at 6 hours, and 10 and 100 ng/mL at 24 and 48 hours. N = 5−6 gels per treatment per time point. *p<0.05 vs. control media, •p<0.001 vs. 1 ng/mL TGF-β1.

Both neotendon transcription factors, Scleraxis (*Scx*) and Mohawk (*Mkx*), were highly upregulated by TGF-β1 in a dose-dependent manner. Increased Mohawk expression was apparent as early as 6 hours after treatment with 1–100 ng/mL of TGF-β1 (Figure 7A), however it only reached significance in the 10 and 100 ng/mL treated gels at 48 hours (p<0.001). Scleraxis was significantly upregulated 3-fold as early as 6 hours after treatment with 100 ng/mL TGF-β1 (p<0.05), and by 4- to 6-fold by 10 and 100 ng/mL TGF-β1 at 24 and 48 hours (p<0.001 - Figure 7B).

### TGF-β1 Shifted the Balance of Gene Expression in Favor of ECM Production

**Figure 8:**
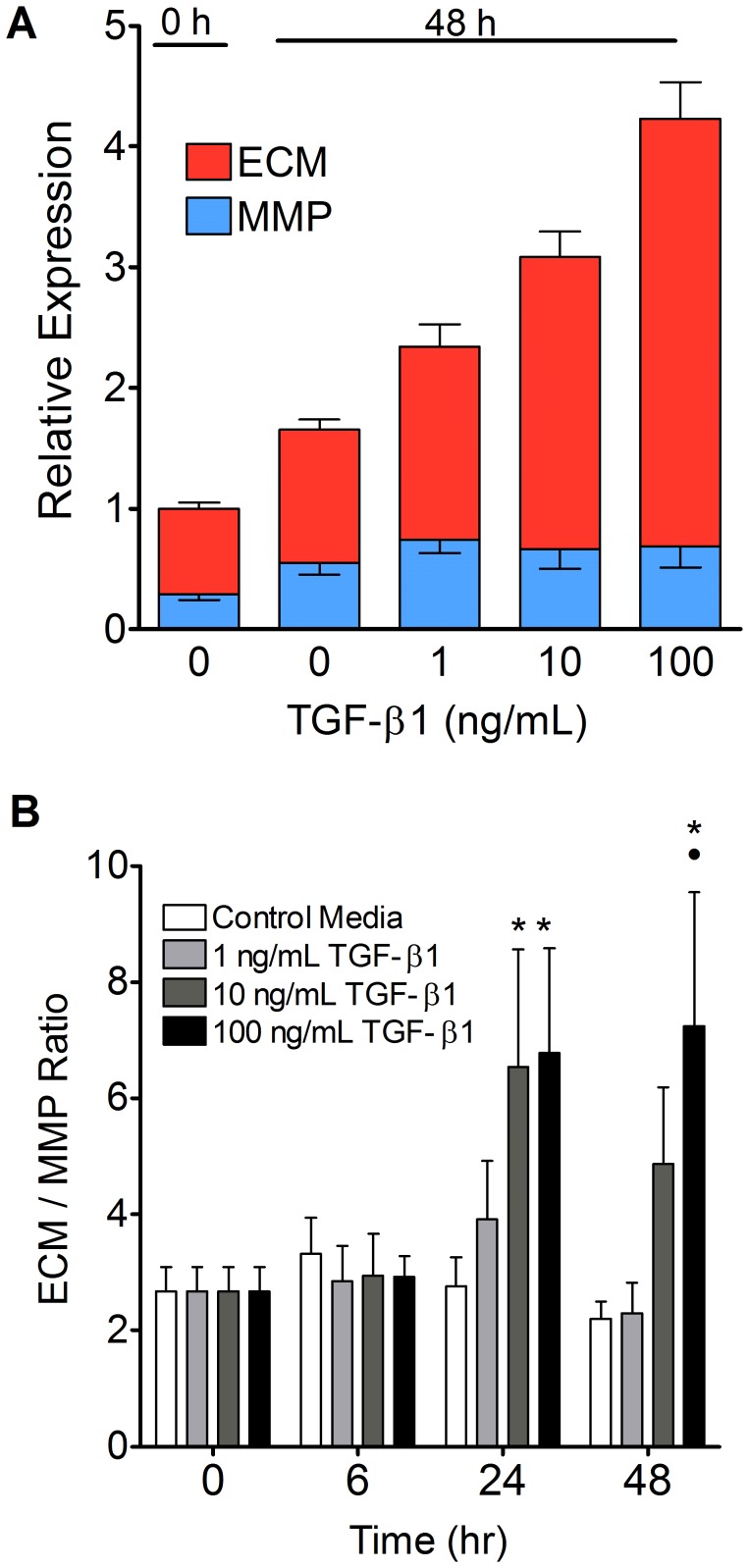
TGF-β1 tilted the balance of ECM and MMP gene expression in favor of ECM. (A) A comparison of the total ECM (includes fibronectin, collagen and proteoglycan genes) vs. total MMP expression (includes MMP-2, -3, -14 and -16 genes) illustrates that TGF-β1 caused dose-dependent increases in overall ECM transcription, but not MMP expression at 48 hours. (B) The ratio of ECM to MMP expression was calculated for each treatment and time point. Gels treated with 10 or 100 ng/mL of TGF-β1 had significantly higher ECM/MMP ratio at 24 hours compared to controls. Gels treated with 100 ng/mL of TGF-β1 also had a significantly higher ECM/MMP ratio at 48 hours. The 1 ng/mL treatment group, however, was not sufficient to alter the ECM/MMP ratio at any time point. *p<0.05 vs. control media, •p<0.01 vs. 1 ng/mL TGF-β1.

An inter-gene analysis was performed to compare the total number of ECM transcripts (i.e. of fibronectin, collagen and proteoglycan genes) with the total number of MMP transcripts (MMP-2, -3, -14 and -16). This analysis showed that higher doses of TGF-β1 caused major increases in ECM expression (red), but not MMP expression (blue) at 48 hours (Figure 8A).

The ratio of expression of ECM to MMP genes was also determined for all treatments and time points (Figure 8B). At 0 hours, there were 2.7 times as many ECM transcripts as MMP transcripts. The ECM/MMP transcript ratio significantly increased to about 6.5 in gels treated with 10 or 100 ng/mL of TGF-β1 at 24 hours (p<0.05). By 48 hours, the ECM/MMP ratio dropped to 2.2 in gels treated with control media or 1 ng/mL of TGF-β1, while gels treated with 10 ng/mL remained elevated at a ratio of 4.9, although this increase was not found to be significant. The ECM/MMP ratio of gels treated with 100 ng/mL of TGF-β1 was 7.2 at 48 hours, remaining significantly higher than gels treated with either control media or 1 ng/mL TGF-β1 (p<0.01).

## Discussion

Using an *in vitro* model of tendon healing, we examined the effects of TGF-β1 on the expression of extracellular matrix proteins, MMPs, MMP inhibitors, and neotendon genes relevant to tendon development, healing and adhesion formation. Our goal was to better characterize the effects of TGF-β1 on tenocyte gene expression, which to date remain mostly not well understood despite some preliminary reports [36], [37], [38], [39], in order to both identify new targets for the prevention of flexor tendon adhesions as well as facilitate future experimentation to understand the molecular underpinnings of TGF-β1’s fibrotic effects. Interestingly, while tenocytes contracted collagen gels to a similar degree after treatment with 1–100 ng/mL of TGF-β1, each level of treatment elicited measurably different responses on gene transcription. In most instances, however, the effects of 10 ng/mL of TGF-β1 on gene expression were similar to those of 100 ng/mL, albeit with some differences in the magnitude and duration of the effect. Nevertheless, the data suggests that 10 ng/mL TGF-β1 is a sufficient dose to elicit the cellular events which caused an increase in fibronectin, collagen, biglycan, PAI-1, Mohawk, and Scleraxis gene transcription as well as MMP-16 and decorin downregulation similar to that which was observed in the 100 ng/mL TGF-β1 gels. We cannot, on the other hand, say the same about the 1 ng/mL dose, which often more closely resembled the control media at a gene transcription level, especially 24 to 48 hours after treatment.

As fibronectin, collagen I, and collagen III are the main components of scar tissue and adhesions, it was expected that TGF-β1 would cause large increases in the expression of these genes. However, we did not expect TGF-β1 to have such a profound effect on collagens V and XII, which are normally associated with the maturation of collagen fibrils during normal tendon development [17]. Similarly, we did not initially expect TGF-β1 to increase the expression of both Scleraxis and Mohawk, transcription factors that play a crucial role in tendon development. Instead, we found that TGF-β1 elicited significant, 6- to 12-fold increases in their expression over control gels. The finding that Scleraxis is upregulated by TGF-β1 in adult tendon cells is consistent with reports that TGF-β1’s role in tissue fibrosis is associated with increased Scleraxis expression in heart [40] and muscle [41]. Therefore, the hypothesis that increased Scleraxis expression is beneficial to tendon healing warrants further investigation. In any case, the finding that TGF-β1 increases Collagen V, XII, Scleraxis and Mohawk expression indicate that TGF-β1 exerts important regenerative effects on tendon healing that must not be neglected. This is consistent with *in vivo* animal models where disrupted TGF-β1 signaling resulted in weaker tendon repairs [8], [9], while over-expression of TGF-β1 was reported to have improved the strength of repairs in a rabbit Achilles tendon healing model [42].

We also examined the effects of TGF-β1 on the expression of biglycan, decorin, and lumican, proteoglycans which play an important role in the development of tendon and regulation of collagen fibrillogenesis (reviewed in [17]). As TGF-β1 is associated with scar tissue formation, we hypothesized that it would inhibit or have little effect on proteoglycan expression. However, TGF-β1 stimulated upregulation of biglycan and downregulation of decorin expression in a dose-dependent manner. TGF-β1 also stimulated a moderate upregulation of lumican expression at 1 and 10 ng/mL, but not at 100 ng/mL. As biglycan is present early in tendon development, but eventually gets replaced by increasing amounts of decorin [43], the balance of proteoglycan expression in healing tendon may be a therapeutic target that warrants further investigation. These findings, taken together with reports that biglycan upregulation is associated with tendinopathy [44], suggest that TGF-β1 may also play an important, but currently undefined role in other conditions that are linked with inflammation [45]. Finally, the inter-gene analysis of proteoglycan expression revealed that biglycan was expressed much more highly than decorin and lumican in the tenocyte-seeded collagen gels. This finding agrees with developmental data that biglycan is most abundant in the early stages of collagen organization, while decorin increases as fibrils mature [43].

Given the important role MMPs play in the turnover of ECM, we hypothesized that TGF-β1 may promote adhesion formation by inhibiting expression of MMPs or upregulating MMP activity modulators (PAI-1, TIMP-2). In terms of MMP expression, TGF-β1 did not inhibit the transcription of MMP-2, MMP-3 or MMP-14. Interestingly, their expression was upregulated over the course of the experiment in a time-dependent manner. This finding is consistent with the observation that fibroblast-mediated collagen gel contraction is MMP-2 and MMP-3 dependent [46]. Despite the time-dependent upregulation of MMP gene expression, the inter-gene analysis of ECM and MMP expression revealed that 10 and 100 ng/mL of TGF-β1 clearly increased the ratio of ECM gene expression to MMP gene expression. In addition, the upregulation of the MMP activity inhibitor, PAI-1, by all three doses of TGF-β1 as early as 6 hours post treatment is consistent with prior reports implicating PAI-1 modulation of MMP-2 activity, rather than MMP-2 transcriptional downregulation, in TGF-β1 mediated renal fibrosis [47] and provides support to our hypothesis that TGF-β1 causes increased ECM production and decreased ECM turnover, leading to the accumulation and persistence of adhesions. These findings warrant formal investigation of the effects of TGF-β1 on MMP activity in future studies.

MMP-16 expression was downregulated by TGF-β1, a novel finding in this experiment. MMP-16 is a membrane-bound MMP which activates other MMPs and promotes collagen fibril formation during tendon development [19]. This finding suggests that increasing MMP-16 expression may help modulate TGF-β1 mediated healing by directing proper regeneration of the tendon microstructure and/or promoting the degradation of undesirable ECM components, which remains to be formally validated.

Finally, the inter-gene analysis of ECM genes strikingly revealed that the tenocytes seeded in collagen I gels produced very high levels of fibronectin (*Fn1*) and collagen III (*Col3a1*). Given that the vast majority of mature tendon ECM consists of collagen I [32], this appeared to be a counterintuitive observation. However, since fibronectin and collagen III are known to be highly upregulated during tendon healing [16], their high expression in the collagen gel model adds support to the applicability of 3D collagen hydrogels as an *in vitro* model of tendon repair. In addition, the inter-gene analysis of the transcription of ECM proteins and MMPs made it clear that higher doses of TGF-β1 tilted the balance of expression in favor of the ECM genes, a possible means by which TGF-β1 contributes to adhesion formation. This novel inter-gene analysis methodology has immense potential to lend valuable insights into countless gene expression experiments.

One important limitation of this study was that tenocytes were subjected to the constantly changing microenvironment of contracting collagen gels. While the changing microenvironment is a potential confounding variable, the data suggests that it did not have a strong effect on gene expression. Firstly, gels treated with 1 ng/mL of TGF-β1 contracted to a similar extent as gels treated with 10 or 100 ng/mL at all time points. The contraction data therefore suggest that gels treated with 1–100 ng/mL TGF-β1 had similar microenvironments throughout the experiment, and that their microenvironments may have differed from the control gels as the gels were remodeled. Therefore, if differences in the microenvironment had a large effect on gene expression, this would cause gels treated with 1 ng/mL to more closely resemble the 10 and 100 ng/mL treatment groups, rather than the control group (as was the case in terms of gel contraction). However, in almost every gene analyzed, the 1 ng/mL gels most closely resembled the control gels at 24 and 48 hours when contraction was most pronounced, suggesting that changes in the microenvironment of the gels did not have a large effect on gene expression throughout duration of the experiment.

There are several other limitations to this study. One was that the tendon-derived cells, which we have thus far referred to as “tenocytes”, are likely a mixed population of cells including epitenon and endotenon fibroblasts, tendon progenitor/stem cells (TSCs), and vascular-associated cells [48], [49]. As the vast majority of tendon cells used for this study exhibited the elongated, fibroblast-like morphology typical of tenocytes, we have referred to them as such. Another limitation of this study was that the effects of mechanical stress variations within the pinned collagen gels were not evaluated. It is known that mechanical forces accumulate throughout the process of cell-mediated collagen gel contraction [50]. In our model, it is likely that tenocytes contracting the gels around the screws experienced compressive forces while tenocytes between the screws were in tension during gel contraction. As both the outer and inner parts of the gel were pooled for RNA analysis, their combined expression was assessed in this study. It is important to note, however, that the geometry of the pinned collagen gels was such that the vast majority of tenocytes were between the screws and therefore experiencing tension.

As our goal was to evaluate the effects of TGF-β1 on gene expression, an important limitation of this study was that protein levels beyond the transcription level were not assessed. We also did not evaluate the signaling pathways involved with TGF-β1 signal transduction such as the Smad [51], [52], [53] and non-Smad pathways [54], [55]. Therefore, future studies are warranted to examine the posttranscriptional regulation of ECM and MMP-related genes in tenocytes as well as the signaling pathways through which TGF-β1exerts its pro-scarring effects.

In conclusion, flexor tendon healing is a complex clinical challenge that requires a detailed understanding of the numerous factors that are involved in complications associated with tendon repairs; namely, inferior repair strength and the formation of debilitating adhesions. Our analysis of TGF-β1’s effects on flexor tendon tenocytes not only provided insights into the positive effects of TGF-β1 on tendon regeneration, it also confirmed the unavoidable fibrotic effects of this factor in terms of tilting the balance of ECM and MMP expression in favor of the former, upregulating the expression of the MMP activity inhibitor, PAI-1, and downregulating the expression of MMP-16. Future studies are warranted to functionally define the role of MMPs in this model and further understand the implications of our findings to the problems associated with flexor tendon healing.

## Materials and Methods

### Ethics Statement

All animals (C57BL/6 mice) used in this study were cared for in accordance with an animal use and care protocol approved by the University Committee on Animal Research (UCAR) of the University of Rochester. Mice were used solely for tendon harvest to isolate tenocytes from flexor tendons of the hind paws. In brief, mice were sacrificed in approved CO_2_ euthanasia chambers and death was verified using cervical dislocation. No live mice were used in this study.

### Tissue Harvest and Cell Culture

Flexor digitorum longus tendons were obtained from the hind limbs of five freshly sacrificed, 7 month old C57BL/6 mice. Specimens were stripped of surrounding tissue, washed in DPBS (Gibco, #14190) and 1% Pen Strep (Gibco, #15140), minced into 1 mm pieces, and trypsinized for 1 hour at room temperature under sterile conditions. The tendon fragments were then cultured in MEM α (Gibco, #12561) supplemented with 20% FBS (Sigma-Aldrich, #F6178), 1% Pen Strep, and 6.5 µL/L of 2-Mercaptoethanol (Sigma-Aldrich, #M7522). The cells that emerged were serially passaged five times and then aliquots of 7 million cells were cryopreserved at −80°C in 50% MEM α, 40% FBS and 10% DMSO (Sigma-Aldrich, #D2650). The tendon cells were later thawed, plated, expanded and used at passage 7 for each of the experiments. As the cells at passage five overwhelmingly exhibited an elongated, fibroblast-like morphology, they were termed “tenocytes” [48].

### Collagen Gel Model of Tendon Healing

The day before each experiment, near-confluent tenocytes were trypsinized for 10 minutes in 0.25% Trypsin-EDTA (Gibco, #25200) in a humidified incubator (5% CO_2_, 37°C). Cells were then washed in culture media, centrifuged, strained to ensure a single cell suspension, and counted using a hemocytometer. The cells were then pelleted, resuspended in control media (MEM α supplemented with 1% FBS and 1% Pen Strep), and mixed with an isotonic, neutral collagen I solution (Advanced BioMatrix, #5005-B) at a volume ratio of 1∶19 to achieve a final cell density of 7×10^5^ cells/mL and collagen concentration of 2.3 mg/mL. The cell-seeded collagen was then cast into custom-made silicone constructs (Figure 1A) around two screws made of Polyetheretherketone (SmallParts, #B000MN4SAI), an autoclave-ready polymer, and gelled in an oven set to 37°C for 1 hour.

After gelation, the edges of the collagen gels were separated from the sides of the silicone construct with a sterile spatula, and 2 mL of control media was added to the media well of each construct (Figure 1A). The following day, the control media was replaced with fresh control media supplemented with 0, 1, 10, or 100 ng/mL of TGF-β1 (R&D Systems, #240-B). A set of gels from each treatment group (n = 3) was imaged immediately before treatment (0 hours) and again at 6, 24 and 48 hours after treatment with a digital camera (SPOT RT3). In addition, a set of gels from each treatment and time point (n = 3) were frozen at -80°C for RNA purification at a later time. At the conclusion of the experiment, images of the gels were analyzed with ImageJ (available at http://rsb.info.nih.gov/ij; developed by Wayne Rasband, National Institutes of Health, Bethesda, MD) to compute area contraction, which was determined by dividing the area of the gel at each time point by its area at 0 hours. The experiment was duplicated for a total sample size of 6 per treatment per time point.

### RNA Purification and Reverse-Transcription

Tenocyte-seeded collagen gels treated with control media supplemented with 0, 1, 10 or 100 ng/mL of TGF-β1 were collected at 0, 6, 24 and 48 hours and frozen at −80°C for RNA purification and reverse-transcription. Within two weeks of each experiment, gels were thawed and vortexed briefly in 1 mL of TRIzol (Ambion, #15596) and RNA was extracted using a modified version of the TRIspin method [56]. Namely, the manufacturer’s protocol for TRIzol extraction was followed until removal of the aqueous layer. The aqueous layer was then mixed with 70% ethanol/RNase free water (Gibco, #10977) and further purified using a mini spin column kit (Epoch Life Science, #1660050) according to the manufacturer’s recommendations. As two samples from an experiment were lost due to a pipetting error during RNA purification, the 10 and 100 ng/mL TGF-β1 treatment groups at 48 hours had an n = 5, while the other groups had an n = 6.

Immediately after purification, the quantity and purity of each sample was measured with a spectrophotometer (NanoDrop 1000). Subsequently, 800–1000 ng of RNA from each gel was reverse-transcribed to cDNA using the iScript cDNA Synthesis Kit (BioRad, #170-8891), and samples were stored at −20°C for gene expression analysis with RT-PCR.

### Real-Time Polymerase Chain Reaction (RT-PCR)

Validated primer sequences for all of the genes analyzed were obtained from PrimerBank [57] and validated with NCBI’s Primer-BLAST for specificity to the desired genes (Table 1). The expression of each collagen isoform was evaluated based on the expression of their alpha 1 chain. For example, collagen I was assessed by the expression of *Col1a1*, and so forth. After obtaining the primers (Integrated DNA Technologies), they were reconstituted to 100 µM in molecular grade water (Gibco, #10977), aliquoted, and stored at −20°C.

**Table 1:** Primers used for RT-PCR analysis of gene expression.

Gene	Forward Primer (5′-3′)	Reverse Primer (5′-3′)	Amplicon Length
Biglycan *(Bgn)*	TGCCATGTGTCCTTTCGGTT	CAGGTCTAGCAGTGTGGTGTC	112
Collagen, type I *(Col1a1)*	GCTCCTCTTAGGGGCCACT	CCACGTCTCACCATTGGGG	103
Collagen, type III *(Col3a1)*	ACGTAGATGAATTGGGATGCAG	GGGTTGGGGCAGTCTAGTG	154
Collagen, type V *(Col5a1)*	TGAGTCTGGTTTTCCCGAGGA	GCCCTGCTCATTGTAAATGGAGA	97
Collagen, type XII *(Col12a1)*	AAGTTGACCCACCTTCCGAC	GGTCCACTGTTATTCTGTAACCC	111
Decorin *(Dcn)*	AAGCTGCGGAAATCCGACTTC	CCCAGAGTTTTTCAGTGGGTTG	81
Eukaryotic translation elongation factor 1 alpha 1 *(Eef1a1)*	TACGCCTGGGTCTTAGACAAA	TCCACAGGGAGATGTCAATAGT	70
Fibronectin (*Fn1*)	GGAGGAAGCCGGGGTTTTAAC	GCGCTCATAAGTGTCACCCA	105
Lumican *(Lum)*	CTCTTGCCTTGGCATTAGTCG	GGTCATCACAGTACATGGCAGT	147
Mohawk (*Mkx*)	CACCGTGACAACCCGTACC	GCACTAGCGTCATCTGCGAG	73
Matrix Metalloproteinase-2 *(Mmp2)*	CAAGTTCCCCGGCGATGTC	TTCTGGTCAAGGTCACCTGTC	171
Matrix Metalloproteinase-3 *(Mmp3)*	ACATGGAGACTTTGTCCCTTTTG	TTGGCTGAGTGGTAGAGTCCC	192
Matrix Metalloproteinase-14 *(Mmp14)*	CAGTATGGCTACCTACCTCCAG	GCCTTGCCTGTCACTTGTAAA	119
Matrix Metalloproteinase-16 *(Mmp16)*	TTACTCGCATTCAGCTCTGGA	CCGCAGACTGTAGCACATAAAA	101
Scleraxis (*Scx*)	CTGGCCTCCAGCTACATTTCT	GTCACGGTCTTTGCTCAACTT	237
Plasminogen Activator Inhibitor 1 *(Serpine1)*	TTCAGCCCTTGCTTGCCTC	ACACTTTTACTCCGAAGTCGGT	116
Tissue Inhibitor of Metalloproteinase 2 *(Timp2)*	TCAGAGCCAAAGCAGTGAGC	GCCGTGTAGATAAACTCGATGTC	142

Triplicate measurements of transcripts from each cDNA sample were performed using the PerfeCTa SYBR Green FastMix (Quanta Biosciences) according to the manufacturer’s protocol. Briefly, 5 µL of cDNA diluted to 1 ng/µL was combined with 15 µL of a 1∶2 mixture of primers (1.2 µM) and SYBR Green FastMix. The expression of target genes was measured using either the Rotor-Gene Q (Qiagen) or Rotor Gene 6000 (Corbett Research) RT-PCR systems.

### Single Gene Analysis of RT-PCR Data

The expression of target genes was normalized to the housekeeping gene, *Eef1a1*, or eukaryotic translation elongation factor 1 alpha 1 [58], which we found to be expressed more uniformly across the various treatments and time points than the other housekeeping genes tested (β-actin, GAPDH and 18s [data not shown]). The efficiency of the RT-PCR reaction for each gene was calculated based on the rate of increase in absolute fluorescence of each sample using the following equation which has been previously described [59]:
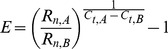
(1)


Here, *E* is the reaction efficiency, *R_n,A_* and *R_n,B_* are two user-defined fluorescence thresholds, and *C_t,A_* and *C_t,B_* are the number of cycles each sample required to reach the fluorescence thresholds, *R_n,A_* and *R_n,B_*, respectively (i.e. Cycles to Threshold). To compensate for variations in the sensitivity of the RT-PCR machine’s detector, thresholds A and B were selected relative to the maximum fluorescence observed at the conclusion of 40 cycles for each gene. Specifically, thresholds were selected to be 3% and 6% of the maximum relative fluorescence of each gene.

After calculating the reaction efficiency of each gene, C_t_ values from the 6% threshold were used to quantify gene expression for the samples according to the following equation, the derivation and validation of which has been described previously [60].
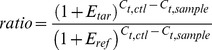
(2)


Where *ratio* refers to the expression of a target gene (*tar*) normalized to the control group of the experiment (*ctl*) and the reference/housekeeping gene (*ref*). *E_tar_* and *E_ref_* are the efficiencies of the target and reference genes, respectively, and *C_t,ctl_* and *C_t,sample_* are the average C_t_ of the control group and the C_t_ of the sample, respectively, for the target gene (in the numerator) and reference gene (in the denominator).

### Inter-Gene Analysis of RT-PCR Data

In addition to examining the response of individual genes to TGF-β1, we also developed a novel mathematical analysis for comparing transcript levels between genes that takes into account the primer-specific differences in amplicon sizes. The analysis is based on the assumption that the fluorescence detected by the RT-PCR machine is proportional to the amount of double-stranded DNA (dsDNA) present after each cycle. Primers that code for longer amplicons would therefore have more dsDNA at any given cycle than primers coding for shorter amplicons, and this varies proportionally with amplicon length according to the following relation:
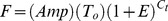
(3)


Where *F* is the fluorescence threshold, *Amp* is the amplicon length, *T_o_* is the initial number of transcripts, *E* is the efficiency of the RT-PCR reaction, and *C_t_* is the cycles to the fluorescence threshold. This equation is almost identical to that described previously [59] with the exception of the additional variable for amplicon size.

As mentioned previously, we used fluorescence thresholds for each gene that were 6% of the maximum relative fluorescence after 40 cycles. Since all genes were assigned the same relative fluorescence threshold, we set the fluorescence of gene x (*F_x_*) equal to that of gene y (*F_y_*) from equation (3) and obtain the following:
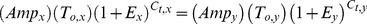
(4)


Rearranging and solving for the ratio of the original number of transcripts of genes x and y, we obtain the following equation:
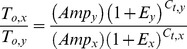
(5)


This equation allows us to compare the transcript levels of different genes within individual samples. We can validate this equation by applying it to the traditional comparison of a sample and control group expressing a single target gene (i.e. amplicon size and efficiency are the same). By doing so, we obtain the following:
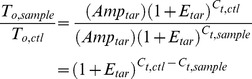
(6)


The right-hand side of the equation is identical to the numerator of equation (2). By normalizing to (i.e. dividing by) the analogous equation for the housekeeping gene, equation (2) is recovered.

Using equation (5), we characterized the relative transcription levels of ECM and MMP genes that are major players in tendon development and healing in tenocyte-seeded collagen gels before and 48 hours after treatment with TGF-β1.

### Statistical Analysis

Differences between treatment groups at each time point were determined for area contraction and RT-PCR data using a two-way ANOVA with Bonferroni post tests in Prism (GraphPad Software). In all cases, a p-value of <0.05 was considered to be significant.
